# UV Differentially Induces Oxidative Stress, DNA Damage and Apoptosis in BCR-ABL1-Positive Cells Sensitive and Resistant to Imatinib

**DOI:** 10.3390/ijms160818111

**Published:** 2015-08-05

**Authors:** Ewelina Synowiec, Grazyna Hoser, Katarzyna Wojcik, Elzbieta Pawlowska, Tomasz Skorski, Janusz Błasiak

**Affiliations:** 1 Department of Molecular Genetics, University of Lodz, Pomorska 141/143, 90-236 Lodz, Poland; E-Mails: ewelinas@biol.uni.lodz.pl (E.S.); katarzyny.wojcik@gmail.com (K.W.); 2 Department of Clinical Cytobiology, Medical Center for Postgraduate Education, Marymoncka 99, 01-813 Warsaw, Poland; E-Mail: graho@cmkp.edu.pl; 3 Department of Orthodontics, Medical University of Lodz, Pomorska 251, 92-216 Lodz, Poland; E-Mail: elzbieta.pawlowska@umed.lodz.pl; 4 Department of Microbiology and Immunology, School of Medicine, Temple University, Philadelphia, PA 19140, USA; E-Mail: tskorski@temple.edu

**Keywords:** BCR-ABL1, imatinib resistance, DNA damage, apoptosis, reactive oxygen species

## Abstract

Chronic myeloid leukemia (CML) cells express the active BCR-ABL1 protein, which has been targeted by imatinib in CML therapy, but resistance to this drug is an emerging problem. BCR-ABL1 induces endogenous oxidative stress promoting genomic instability and imatinib resistance. In the present work, we investigated the extent of oxidative stress, DNA damage, apoptosis and expression of apoptosis-related genes in BCR-ABL1 cells sensitive and resistant to imatinib. The resistance resulted either from the Y253H mutation in the BCR-ABL1 gene or incubation in increasing concentrations of imatinib (AR). UV irradiation at a dose rate of 0.12 J/(m^2^·s) induced more DNA damage detected by the T4 pyrimidine dimers glycosylase and hOGG1, recognizing oxidative modifications to DNA bases in imatinib-resistant than -sensitive cells. The resistant cells displayed also higher susceptibility to UV-induced apoptosis. These cells had lower native mitochondrial membrane potential than imatinib-sensitive cells, but UV-irradiation reversed that relationship. We observed a significant lowering of the expression of the succinate dehydrogenase (*SDHB*) gene, encoding a component of the complex II of the mitochondrial respiratory chain, which is involved in apoptosis sensing. Although detailed mechanism of imatinib resistance in AR cells in unknown, we detected the presence of the Y253H mutation in a fraction of these cells. In conclusion, imatinib-resistant cells may display a different extent of genome instability than their imatinib-sensitive counterparts, which may follow their different reactions to both endogenous and exogenous DNA-damaging factors, including DNA repair and apoptosis.

## 1. Introduction

Imatinib (imatinib mesylate) was the first successfully applied drug of targeted anti-cancer therapy [1]. It represents the first generation of tyrosine kinases inhibitors (TKIs) used in chronic myeloid leukemia (CML) and other cancers, including acute lymphoblastic leukemia, gastrointestinal stromal tumor [2]. Imatinib inhibits the activity of the BCR-ABL1 tyrosine kinase, a hallmark of CML, by blocking binding of its co-factor and inducing apoptosis in CML cells. Despite therapeutic success of imatinib, its resistance has become an emerging problem, which has been resolved only in part by second- and third-generation TKIs [3]. Imatinib resistance may be broadly categorized into primary, underlined by mutations in the *BCR-ABL1* gene, and secondary or acquired resistance following imatinib treatment. Several mechanisms associated with the *BCR-ABL1* gene can underline imatinib-resistance, including *BCR-ABL* amplification, its mutations and epigenetic modifications as well as interference with BCR-ABL1-signaling [4]. However, detailed pathways leading to imatinib-resistance are not exactly known.

CML, similarly to other cancers, is characterized by genomic instability, which, at least in part, is induced by the BCR-ABL1 kinase. The kinase can stimulate the production of reactive oxygen species (ROS), which damage DNA and induce cellular redox imbalance [5]. Such endogenous oxidative stress may promote increased susceptibility to exogenous oxidative stress induced by environmental factors, including UV light. ROS-induced DNA lesions can be misrepaired by mechanisms with the involvement of BCR-ABL1 [6,7]. Therefore, BCR-ABL1 may induce DNA damage, contributing to genomic instability, which is then further increased by the BCR-ABL1-dependent mechanism of these damages. We previously showed that BCR-ABL1-induced genomic instability might be associated not only with cancer phenotype of BCR-ABL^+^ cells, but also with imatinib-resistance [8]. Genomic instability is mainly determined by cellular DNA damage response (DDR), in which DNA repair plays a pivotal role. We showed that BCR-ABL1 modulated DNA repair in many kinds of cells [9,10,11,12,13]. As BCR-ABL1 contains redox-sensitive cysteine residues, exogenous ROS can change the structure of this protein leading to alterations in its interaction with small molecules, which may eventually result in imatinib resistance [14].

Main UV-induced DNA damages are 2,3-cyclobutane pyrimidine dimer and pyrimidine (6-4) pyrimidone photoproduct, which in humans are processed by nucleotide excision repair [15]. However, UV may induce a variety of other damages, which can result from its stimulation of ROS production and lead to apoptosis [16]. It was shown that UV-induced ROS production was associated with decreased mitochondrial potential [17]. Therefore, cellular reaction to UV damage may involve essentially the same components, which may be associated with imatinib-resistance in CML cells: nucleotide excision repair as the most versatile system of DNA repair, playing a pivotal role in the maintenance of genomic stability, ROS neutralization, apoptosis and mitochondrial functioning. Therefore, in searching for the mechanism underlying difference between imatinib-resistant and -sensitive cells, it is reasonable to check some components of DNA damage response (DDR) in these cells. In the present work, we investigated UV-induced DNA damage and its repair, apoptosis, ROS production and the expression of *SDHB* (succinate dehydrogenase complex, subunit B), *MCL-1* (myeloid cell leukemia sequence 1), mitochondrial *MT-COX1* (cytochrome c oxidase subunit I), *MT-ND3* (NADH dehydrogenase subunit 3) and *MT-CYTB* (cytochrome B) genes in cells sensitive and resistant to imatinib. These genes are mainly involved in metabolic/respiratory processes, which are associated with ROS production and they all can be associated with apoptosis, although they are not key players in this process.

## 2. Results

### 2.1. Cell Viability after Imatinib Treatment

**Figure 1:**
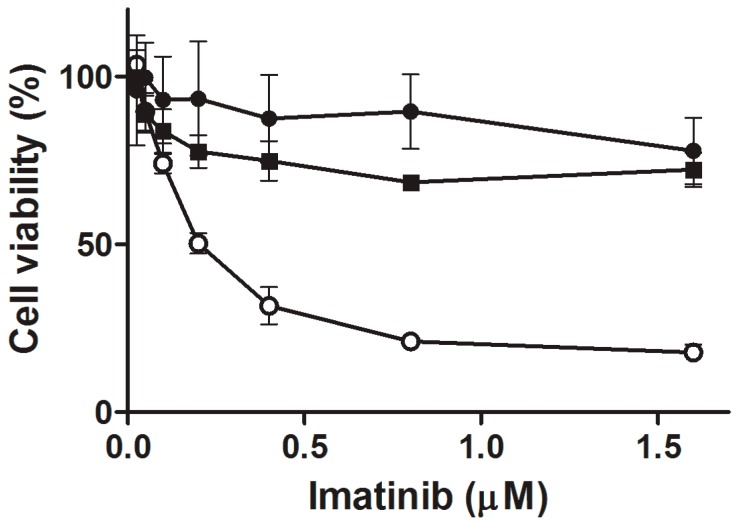
Relative mean viability of mouse 32D cells transfected with the *BCR-ABL1* gene: sensitive (S, white circles) and resistant to imatinib as evaluated by the Cell Counting Kit-8 assay. Imatinib resistance resulted from the Y253H mutation in the *BCR-ABL1* gene (black circles) or from incubation of S cells with escalating doses of imatinib (black squares). All cell lines were incubated with imatinib at 0.025–1.6 µM for 24 h at 37 °C. Presented are means of six independent measurements; error bars represent standard deviation (SD).

All cell lines were incubated for 24 h with various imatinib concentrations. The viability of imatinib-resistant cells harboring the Y253H (253) mutation and with acquired resistance (AR) cells did not change after the incubation, but the subline with non-mutated *BCR-ABL1* (S) decreased its viability to about 17% (Figure 1). Therefore, we considered further the S subline as imatinib-sensitive, whereas 253 and AR sublines were considered as imatinib-resistant.

### 2.2. Cells Primary Resistant to Imatinib Produce More ROS both Endogenously and Following UV Exposure

**Figure 2:**
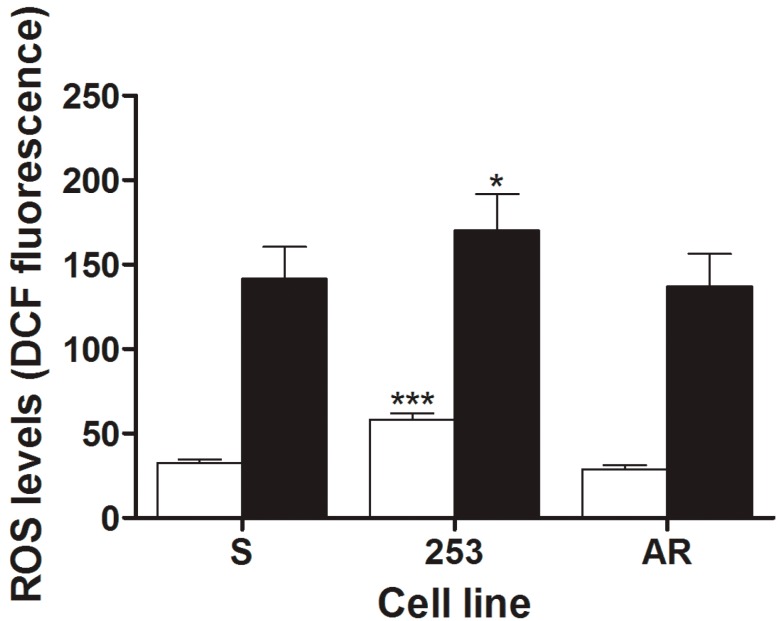
Mean intracellular reactive oxygen species levels in mouse 32D cells transfected with the *BCR-ABL1* gene: sensitive (S) and resistant to imatinib expressed as the fluorescence of 2′,7′-dichlorofluorescein (DCF) oxidatively converted from dichlorodihydrofluorescein diacetate exposed to UV radiation at 35 J/m^2^ at room temperature at a dose rate of 0.12 J/(m^2^·s) (black bars) as compared with non-irradiated cells (white bars). Imatinib resistance resulted from the Y253H mutation in *BCR-ABL1* (253) or from incubation of S cells with escalating doses of imatinib (AR). Presented are means of six independent measurements; error bars represent SEM; * *p* < 0.05, *** *p* < 0.001 as compared with S line.

Endogenous ROS levels in 253 imatinib-resistant cells were higher than in their sensitive counterparts (*p* < 0.001) (Figure 2). UV radiation induced an evident increase in ROS level in all cell lines, but ROS production in 253 cells was still higher than in S line (*p* < 0.001). Therefore, primary imatinib-resistant cells accommodated more ROS than cells with acquired imatinib resistance, which might contribute to genome instability of these cells.

### 2.3. Imatinib-Resistant Cells Have More Pyrimidine Dimers and Oxidative DNA Damage Induced by UV

**Figure 3:**
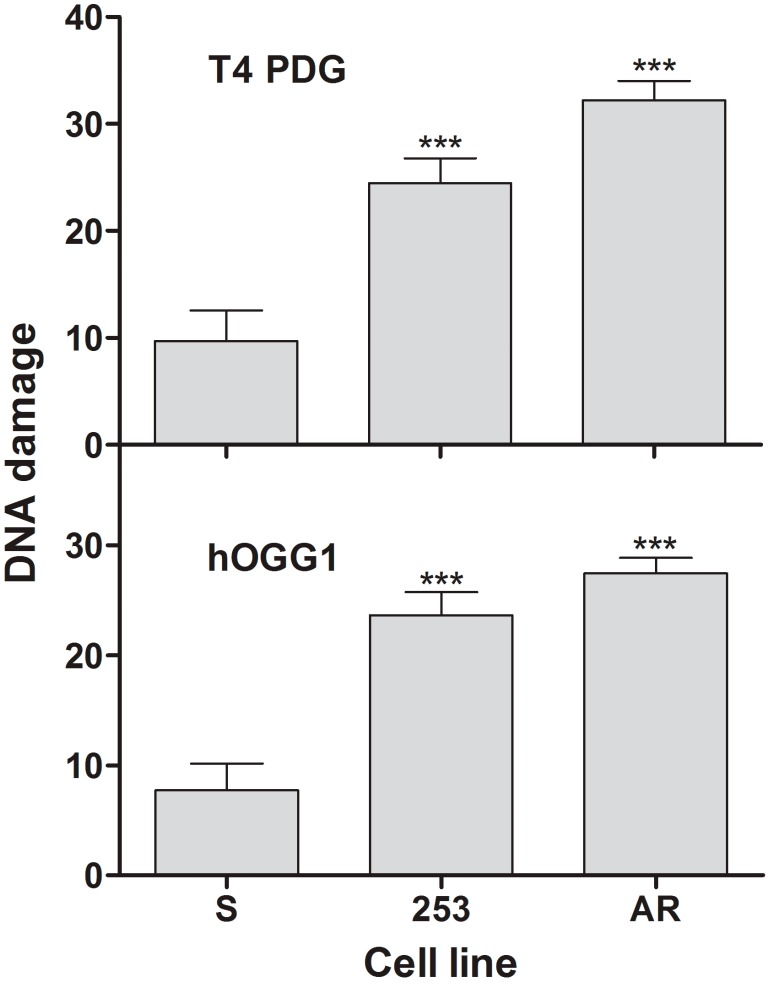
Mean DNA damage measured by percentage of DNA in tail of comets in alkaline version of comet assay modified by the use of T4 pyrimidine dimer glycosylase (T4 PDG) and human 8-oxoG glycosylase (hOGG1) in mouse 32D cells transfected with the *BCR-ABL1* gene: sensitive (S) and resistant to imatinib exposed to UV radiation at 35 J/m^2^ at room temperature at a dose rate of 0.12 J/(m^2^·s). Imatinib resistance resulted from the Y253H mutation in *BCR-ABL1* (253) or from incubation of S cells with escalating doses of imatinib (AR). Presented are means of three independent measurements, each for 100 cells; error bars represent SEM; *** *p* < 0.001 as compared with S line.

UV light induced DNA damage recognized by T4 pyrimidine dimer glycosylase (T4 PDG) and hOGG1 in all kinds of cells (Figure 3). Therefore, each cell line accommodated pyrimidine dimers and oxidative base modifications, including 8-oxoG. However, different sensitivity to these UV-induced products was observed in different cell lines. For both enzymes, the extent of DNA damage in imatinib-resistant lines was much more pronounced that in their sensitive counterparts (*p* < 0.001). There was not any significant difference in the extent of DNA damage induced by either enzyme between Y253H and AR cells. This suggests that imatinib-resistant cells present different DNA damage response than their imatinib-sensitive counterparts. As we showed previously, BCR-ABL1 stimulated DNA repair in imatinib-sensitive cells [9,10,11,12], but imatinib-resistant cells may not follow that mechanism due to a higher level of genome instability.

### 2.4. Imatinib-Resistant Cells Are More Prone to UV-Induced Apoptosis

**Figure 4:**
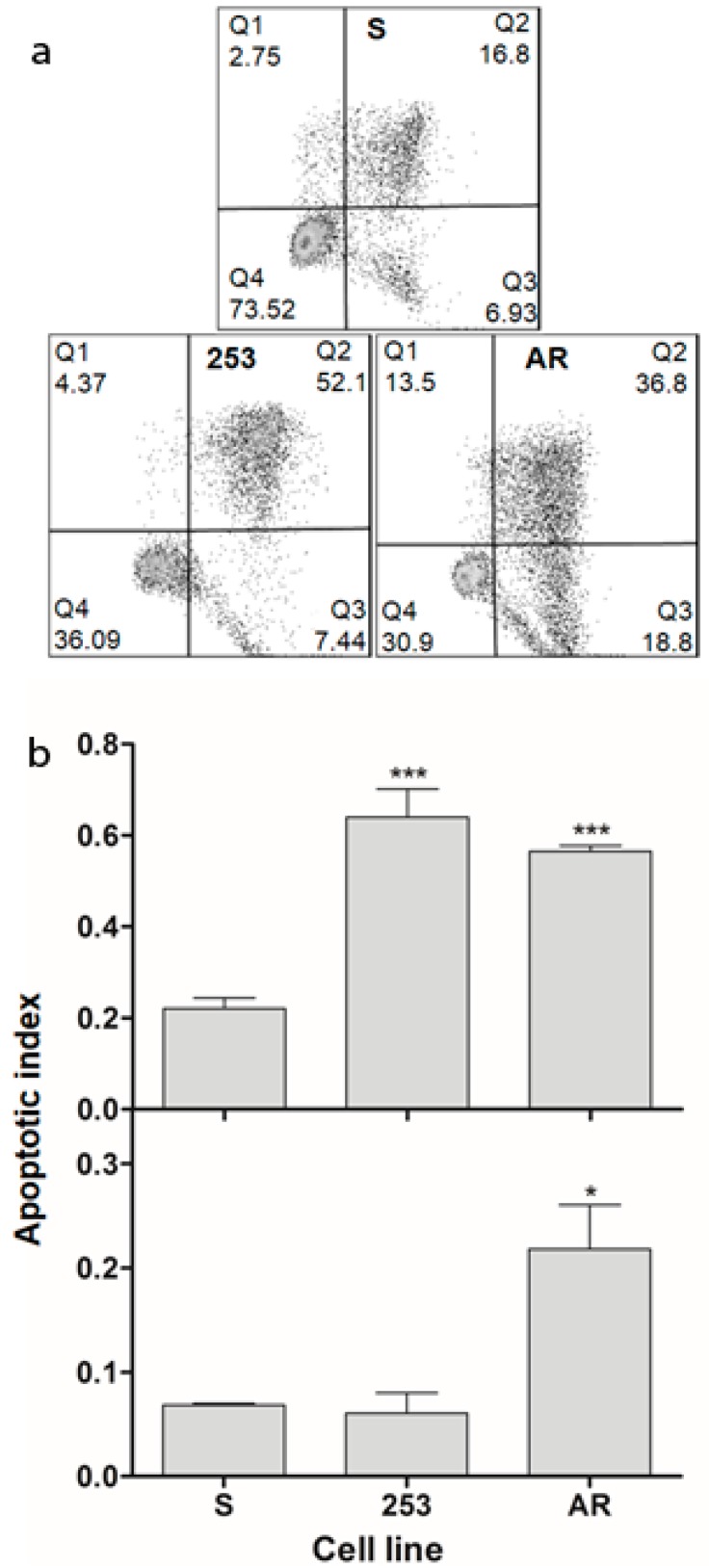
UV-induced apoptosis in mouse 32D transfected with the *BCR-ABL1* gene: sensitive (S) and resistant to imatinib or cells displaying imatinib resistance caused by the Y253H mutation in *BCR-ABL1* (253) or incubation of the sensitive cells with increasing doses of imatinib (AR). Cells were irradiated with UV light at 35 J/m^2^ at room temperature at a dose rate of 0.12 J/(m^2^·s). Apoptosis was assayed by flow cytometry with Annexin V-propidium iodide staining after a 24 h post-incubation. (**a**) Representative scatter plots for cells irradiated with UV; numbers in quadrants represent percentage of cells in early apoptosis (Q3), late apoptosis and necrosis (Q2) and live cells (Q4); (**b**) Apoptotic index calculated as percentage of either Q3 + Q2 cells (upper bar graph) or Q3 cells (lower graph) cells in 5 × 10^4^ cells. Data are expressed as means of three independent experiments, presented is mean ± standard deviation (SD), * *p* < 0.05, *** *p* < 0.001 as compared with S cells.

UV-irradiation evoked apoptosis in all cell sublines (Figure 4). The apoptotic index of S cells was lower than all remaining sublines. This quantity measured as the fraction of only early apoptotic cells was significantly higher in AR cells than S and 253 cells. Therefore, imatinib-resistant cells were more prone to apoptosis than their imatinib-sensitive counterparts. It may seem to be surprising as imatinib resistance brings resistance to apoptosis induced by this drug. However, imatinib resistance does not imply resistance to all apoptotic pathways induced by various stimuli.

### 2.5. Imatinib-Resistant Cells Display Lower Mitochondrial Membrane Potential after UV Exposure

**Figure 5:**
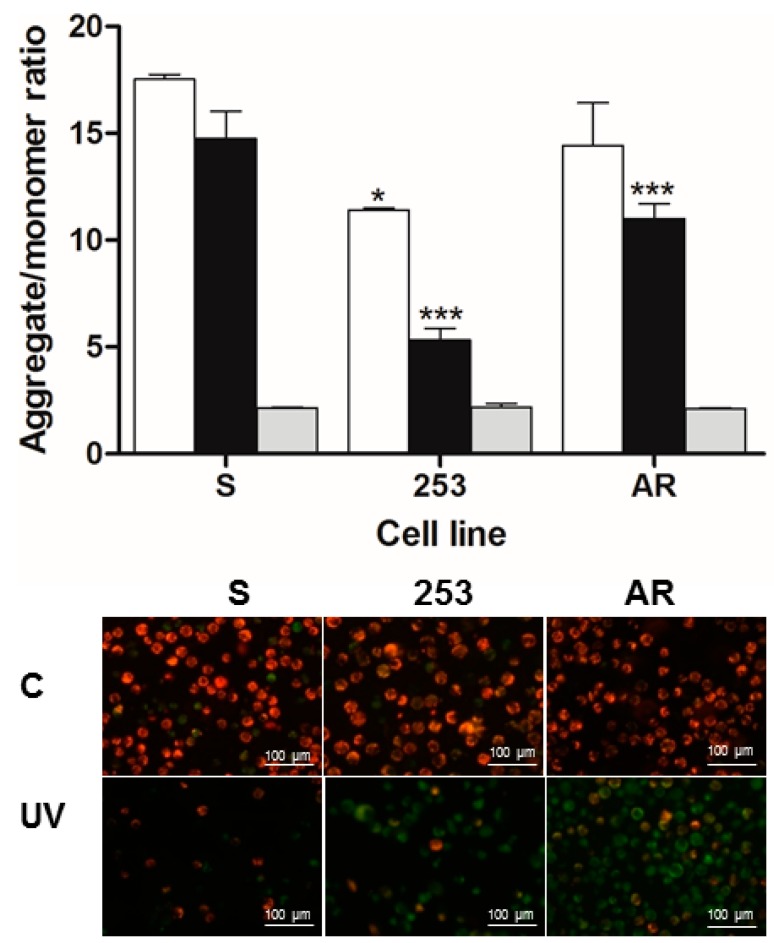
Mitochondrial membrane potential (MMP) in mouse 32D cells transfected with the *BCR-ABL1* gene: sensitive (S) to imatinib or displaying imatinib resistance caused by the Y253H mutation in *BCR-ABL1* (253) or incubation of the sensitive cells with increasing doses of the drug (AR). **Upper** panel. MMP is expressed as ratio of 530 nm/590 nm to 485 nm/538 nm (aggregates to monomer) fluorescence as quantified with a fluorescent plate reader after JC-1 staining. Cells were irradiated with UV light at 35 J/m^2^ at room temperature at a dose rate of 0.12 J/(m^2^·s) (UV, black bars) and compared to control, non-irradiated cells (C, white bars). The mitochondrial uncoupler CCCP (carbonyl cyanide 3 chlorophenylhydrazone) was used as a positive control (grey bars). Fluorescence was measured after a 24 h incubation. Values are means ± SD (*n* = 4). * *p* < 0.05 and *** *p* < 0.001 as compared with imatinib-sensitive cells, respectively; **Lower** panel. Fluorescence microscopy (400×) of control cells (C) and cells treated with UV light (UV). Mitochondria in C cells are polarized and JC-1 accumulates in mitochondria as aggregate with red fluorescence, while in apoptotic cells JC-1 remains in cytoplasm in monomeric form emitting green fluorescence.

All BCR-ABL1-transfected cells displayed lower native MMP than their parental, non-transfected counterparts and both imatinib-resistant cell lines had lower native MMP than S cells (Figure 5). UV irradiation lowered MMP in all kinds of cells. The imatinib-resistant 253 and AR cells displayed significantly lower MMP than imatinib-sensitive S cells. These results are in general agreement with those obtained in flow cytometry study, as membrane mitochondrial potential decreases during apoptosis.

### 2.6. Gene Expression

**Figure 6:**
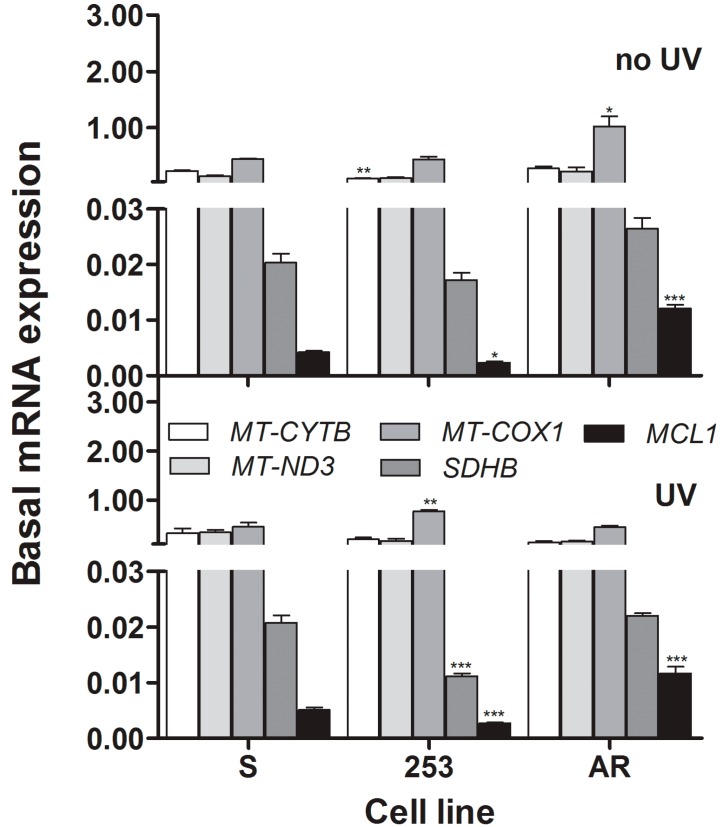
Basal mRNA expression of the nuclear *SDHB* (succinate dehydrogenase complex, subunit B), *Mcl-1* (myeloid cell leukemia sequence 1) and mitochondrial *COX1* (cytochrome c oxidase subunit I) gene in non-irradiated (**upper** panel) or UV-irradiated (**lower** panel) mouse 32D cells transfected with the *BCR-ABL1* gene sensitive to imatinib (S) or cells displaying imatinib resistance caused by the Y253H mutation in *BCR-ABL1* (253) or incubation of the sensitive cells with increasing doses of imatinib (AR). Cells were irradiated with UV light at 35 J/m^2^ at room temperature at a dose rate of 0.12 J/(m^2^·s) and the expression was determined by TaqMan probe-based real-time PCR (RT-PCR) assay and calculated by the 2^−Δ*C*t^ method and Δ*C*_t_ was obtained by subtracting *C*_t_ of ACTB mRNA from *C*_t_ of mRNA of respective nuclear gene or *C*_t_ of *RNR2* from *C*_t_ of *COX1*. Data are shown as means ± SEM (*n* = 3), * *p* < 0.005, ** *p* < 0.01, *** *p* < 0.001 as compared with imatinib-sensitive cells.

We observed some statistically significant differences in basal expression of the MT-CYTB, MT-COX1 and MCL1 mRNAs levels between imatinib-resistant cells and their sensitive counterparts (Figure 6). However, these differences were neither systematic nor pronounced, so we considered them as not clinically relevant. UV radiation did not change the pattern of expression of all genes, and revealed significant and pronounced—at 100%—differences in the expression of the *SDHB* and *MCL-1* genes between resistant and sensitive lines.

### 2.7. A Fraction of AR Cells Have the Y253H, But Not T315I Mutation

**Figure 7:**
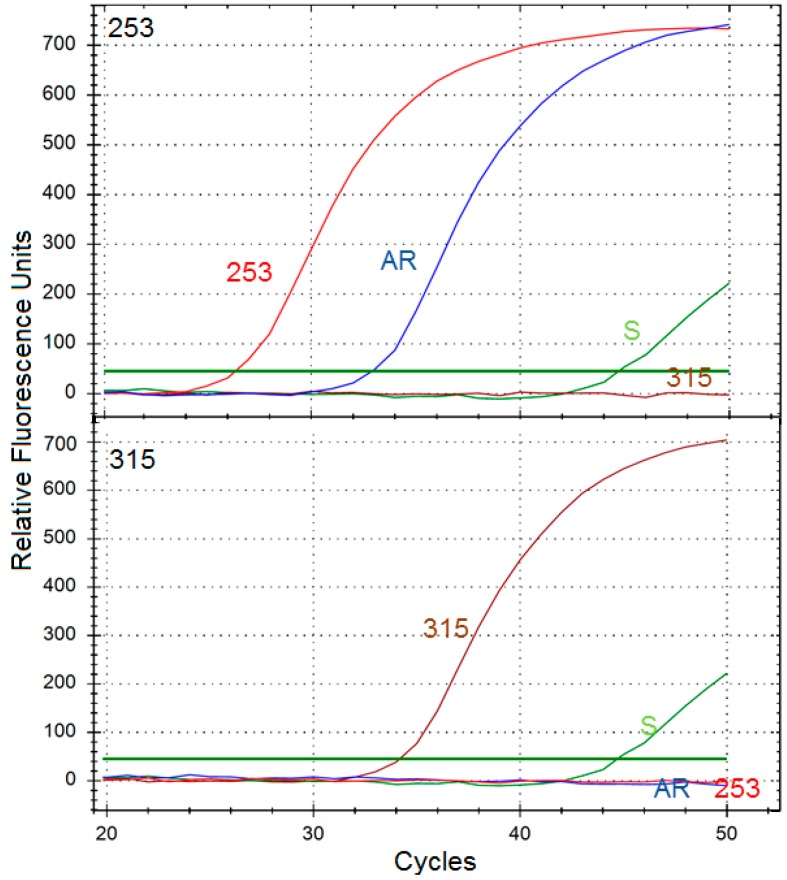
Results of a representative mutational analysis of BCR-ABL1-expressing cells: sensitive to imatinib (S) and primarily resistant to imatinib due to the Y253H with acquired resistance to imatinib. The analysis was based on an allele-specific RT-PCR with primers specific to cDNA obtained by reverse transcription of mRNA produced from the gene of active BCR-ABL1 kinase. The upper plot shows amplification with primers specific for the Y253H mutation, the lower shows those for the T315I mutation. Cell line harboring the T315I mutation (315) and cultured in the same conditions as 253 was used as a positive control.

The results obtained in RT-PCR analysis with primers specific for the Y253H mutation suggest that a part of the AR cell population acquired that mutation (Figure 7). On the other hand, the T315I mutation, frequently found among imatinib-resistant CML patients, was not observed in AR cells. This would explain, at least in part, similar effects of UV radiation in 253 and AR cells, although this similarity was not complete.

## 3. Discussion

Point mutations in the *BCR-ABL1* gene exhibit the most significant factors that cause imatinib resistance. However, they may represent different molecular mechanisms leading to this effect. Therefore, we focused on a single mutation to avoid too many comparisons. The T315I mutation, directly preventing imatinib binding, is probably the most frequent mutation in CML patients and, therefore, it is the most frequently studied. In this work, we focused on the Y253H mutation, representing slightly different mechanism, as it occurs at the nucleotide-binding loop and impairs optimal conformation of this domain of the kinase needed for imatinib binding.

We performed our research on our original CML cell model representing leukemic (BCR-ABL1-expressed) cells sensitive and resistant to imatinib with primary and secondary resistance to the drug. Therefore, our model is homogenous, as derived from a single cell line and strictly defined. At present, we do not have such a human model of CML cells, and using e.g., K562 or LAMA84 cells sensitive and resistant to imatinib would not allow to directly compare the results with those obtained for 32D due to lack of right parental cells for K562 and LAMA84.

In this work, we observed that imatinib-resistant CML model cells displayed different reaction to UV light exposure than their imatinib-sensitive counterparts as they produced more ROS, displayed a higher extent of DNA damage and were more prone to apoptosis induced by UV radiation.

These features may reflect a higher degree of genomic instability of imatinib-resistant cells. We previously showed that BCR-ABL1 stimulated DNA double strand breaks repair by homologous recombination [18]. Recently, it was shown that BCR-ABL1-expressing and imatinib-resistant cell lines and their CML counterparts displayed a higher level of the PARP1 and DNA ligase IIIα proteins crucial for DNA double strand breaks repair in these cells [19]. However, these proteins were involved in an alternative non-homologous end-joining pathway, an error-prone DNA repair system, which might increase genomic instability of imatinib-resistant cells. In our previous work, we postulated that genomic instability of chronic myeloid cells in chronic phase might result from imatinib-resistant CML stem cells [20]. In that context, our present work supports that idea. The problem of genomic instability of CML cells is associated with the BCR-ABL1 kinase, present in these cells. However, CML cell is a broad term, which refers to a heterogeneous population of both stem and progenitor cells, possessing different BCR-ABL1 activities. As the primary cells we used were taken from mouse bone marrow *en masse*, they should be considered as CML progenitor cells. However, the general problem of the involvement of BCR-ABL1 in genomic instability of CML cells is not completely known, and in the present work we do not aimed to evaluate the role of BCR-ABL1 in imatinib-resistance in this work and the results for parental, BCR-ABL1-negative cells were for reference only, not for a direct comparison, and we did not show them. We compared just imatinib-resistant and -sensitive cells. On the other side, manipulating with the BCR-ABL1 activity, for instance by the shRNA technology, would bring too many variables for a reasonable analysis at this stage.

We showed that imatinib-resistant cells harboring the Y253H mutation produced more ROS than sensitive cells and UV greatly potentiated this relationship (Figure 2). As we mentioned, we previously showed a positive correlation between BCR-ABL1, ROS production and imatinib resistance [8]. Therefore, it is not surprising, that UV radiation produced more ROS in imatinib resistant clones, as they might present a lower protective mechanisms against this factor, resulting from their increased genomic instability. There are many pathways of ROS production upon UV irradiation and we did not investigate whether some of them could be altered in imatinib-resistant cells. We speculate that the difference in the extent of ROS between resistant and sensitive cells resulted from deregulated mechanisms of protection against them in resistant cells, following from their increased genomic instability. This was supported by the next experiments showing that UV induced more DNA damage in imatinib-resistant than imatinib-sensitive cells (Figure 3). The major DNA lesions induced by UV are cyclobutane pyrimidine dimers (CPDs) and (6-4)-photoproducts ((6-4)PPs), which are formed rather by a direct photochemical mechanism than by ROS mediators [21]. That is why we chose UV radiation as a DNA-damaging factor to show that the increased genomic instability could affect processing of not only ROS-induced changes. However, the extent of DNA damages we measured resulted from two processes—their induction and repair. CPDs and (6-4)PPs in human cells are primarily repaired by the nucleotide excision repair pathway (NER), whereby proteins can be affected by UV-induced ROS. Therefore, imatinib-resistant cells might have less effective NER than their sensitive counterparts. We previously showed that imatinib reduced NER activity in BCR-ABL1 cells [9]. On the other hand, we also showed that BCR-ABL1 inhibited NER in various human and mouse cells, increasing their sensitivity to UV radiation [22]. It was also shown that genotypes of the single nucleotide polymorphisms 499C>T and 939A>C in the *XPC* gene and their CA haplotype are associated with imatinib response in CML-CP patients [23]. The XPC protein, which is an essential nucleotide excision repair component, is needed for UV-damaged DNA sites to be recognized by two proteins crucial for DNA damage reaction: ATM and ATR [24].

We previously showed that BCR-ABL1 inhibited uracil DNA glycosylase, a base excision repair (BER) enzyme, enhancing oxidative DNA damage and overall genomic instability [13]. On the other hand, two other BER glycosylases, MBD4 (methyl-CpG binding domain protein 4) and NTHL1 (nth (endonuclease III)-like 1), were reported to upregulate in imatinib-resistant K562 cells [25].

Genomic instability and apoptosis are intimately associated phenomena, but this a multifaceted association [26]. If we assume that apoptosis in our experiment was induced by DNA damage evoked by UV radiation, then the extent of this damage was high enough to induce cell cycle arrest, followed by death program directly or with autophagy as an intermediate process [27]. When imatinib-resistant cells represent an increased genomic instability, an excess of DNA damages/mutations may affect DNA repair, cell cycle regulators and apoptosis-related genes. Imatinib is an apoptosis-induced drug, so resistance to it may involve an anti-apoptotic mechanism. Therefore, imatinib-resistant cells seem to be more resistant to apoptosis than their sensitive counterparts, which is in apparent contrast with the results we obtained. However, it is known that imatinib-resistant cells display increased resistance to apoptosis only in the presence of imatinib, and removal of the drug can result in the sensitization of the imatinib-resistant cells to apoptosis-inducing factors [28]. Furthermore, that equivocal linkage between apoptosis and genetic instability does not allow to draw a firm conclusion on the relationship between imatinib-resistant and -sensitive cells in their susceptibility to apoptosis.

The difference in apoptosis susceptibility between imatinib-resistant and sensitive cells was confirmed by the opposite relationship in mitochondrial membrane potential as it is reversely associated with apoptosis progression [28].

Basal MMP in 253 cells was significantly lowered compared to imatinib-sensitive cells (Figure 5). Does this mean that they had a higher basal apoptosis? First, as we mentioned, 253 cells display increased resistance to apoptosis only in the presence of imatinib; Second, changes in MMP may not be directly displayed in viability and apoptosis evaluated in our study. However, this is somehow intriguing and suggests difference between 253 and AR and the involvement of mitochondria in imatinib resistance and definitely requires further studies.

We observed a significant lowering of the expression of the *SDHB* gene in imatinib-resistant cells. This gene encodes the SDHB protein, which is a subunit of the complex II of the mitochondrial respiratory chain containing three more subunits [29,30]. This complex plays an important role in detecting induction of apoptosis and mediates many apoptosis signals. Therefore, observed difference in the expression of the SDHB gene between 253 imatinib-resistant and -sensitive cells may contribute to the increased sensitivity of the mutated cells to UV-induced apoptosis. However, this is apparently not the only reason for this as AR cells also displayed increased sensitivity to apoptosis, a process which is regulated by the expression and interaction of many genes. A significant increase in the MCL-1 mRNA expression in AR cells may be associated with different ant-apoptotic mechanisms of these cells than occurring in their 253 counterparts, in which apoptosis-resistance is stimulated by imatinib. Moreover, this result may confirm that mitochondria are involved in imatinib resistance as mouse MCL-1 localizes in mitochondrial matrix [31]. Our study at this stage provides information on possible direct involvement of metabolic/respiratory and apoptosis-related genes in imatinib-resistance.

We observed that a fraction of AR cells acquired the Y253H mutation during culturing in increasing doses of imatinib. However, the population of AR presented significant imatinib resistance, so we can speculate that the Y253H mutation is only partly responsible for the resistance of AR cells. This may be somehow surprising as Wang *et al.* observed that KCL-22M tyrosine kinase resistant cells acquired the T315I mutation following TKIs exposure [32]. Moreover, this process was also associated with activation of multiple drug resistance pathways. There are several mechanisms of imatinib resistance, including the drug export by α1-acid glycoprotein and its import by hOCT1 protein as well as activation of signaling pathways leading to BCR-ABL1-independent growth, including Ras/Raf/Mek, PI3K, Stat and Erk pathways [33]. We do not exactly know which mechanism underlines the resistance of AR cells, except the Y253H mutation and we did not even try to attempt to determine this mechanism as this would require an additional extensive study. Moreover, we believe that the acquired resistance to imatinib may be underlined by various mechanisms, even it is induced in apparently similar conditions. This is supported by some clinical observations [34].

## 4. Experimental Section

### 4.1. Reagents

Imatinib mesylate was kindly donated by Novartis (Basel, Switzerland). IMDM medium, fetal bovine serum, penicillin, streptomycin and l-glutamine mixture were obtained from Lonza (Basel, Switzerland). Cell Counting Kit-8 assay (CCK-8) was purchased from Sigma-Aldrich (St. Louis, MO, USA). ISOLATE II RNA Mini Kit was purchased from Bioline Reagents Ltd. (London, UK). MitoProbe™ JC-1 Assay Kit, High-Capacity cDNA Reverse Transcription Kit, TaqMan^®^ Genotyping Master Mix and probes were obtained from Life Technologies (Grand Island, NY, USA). Annexin V-FITC Apoptosis Detection Kit I was purchased from BD Pharmingen (Franklin Lakes, NJ, USA).

### 4.2. Cells

This study was performed on CML model cells derived from mouse 32D cell line obtained from bone marrow of the C3H/HeJ mice and transfected with native and mutated p210 BCR-ABL1 as described elsewhere [18]. This resulted in parental 32D cells and three BCR-ABL1-expressing sublines: one sensitive (S) and three resistant to imatinib. Imatinib resistance was underlined by the Y253H mutation in the *BCR-ABL1* gene (253) or was acquired by the incubation of S cells with escalating doses of imatinib (AR). The production of the AR clone started from the addition of imatinib to a final concentration of 0.1 µM to the medium of sensitive cells and replacing it with fresh imatinib solution every 24 h. After reaching a survival rate at the passage not lesser than 80%, which took on average 1–3 weeks, the concentration of imatinib was increased to 0.05. Step by step, with the concentrations of imatinib 0.1, 0.2, 0.3, 0.5, 0.6, 0.8 µM, a clone surviving in at least 80% in medium containing 1.0 µM. Typical procedure leading to the production of AR clone took about 6 months. Then, this clone was treated every 5–10 passage with 0.5 µM imatinib. The parental cells required interleukin 3 to proliferate. It was supplied in the supernatant produced by WEHI-3B cells. In the mutational analysis the cell line with the T315I mutation, frequently found among imatinib-resistant CML patients, was used as a positive control.

### 4.3. UV Irradiation

Cells were UV-irradiated (35 J/m^2^) at room temperature using UVC-6-12 lamp (NeoLab, Heidelberg, Germany) emitting UV light at 254 nm at a dose rate of 0.12 J/(m^2^·s). After irradiation, cells were kept in an incubator with 5% CO_2_ atmosphere at 100% humidity and 37 °C.

### 4.4. Cell Viability

Cell viability was assessed with using the CCK-8 assay. Briefly, cells (5 × 10^4^ per well) were grown in 96-well plates overnight. On the next day, they were incubated with imatinib at 0 (control), 0.025, 0.05, 0.1, 0.2, 0.4, 0.8, 1.6, 3.2, 6.4 µM. After 24 h, the cells were centrifuged (300× *g* for 10 min at 22 °C) and washed with HBSS. Next, a 10 µL aliquot of kit reagent was added to each well and incubated for 3 h at 37 °C. The optical density (OD) was measured at 450 nm with a reference at 650 nm using a Bio-Tek Synergy HT Microplate Reader (Bio-Tek Instruments, Winooski, VT, USA). Cell viability was expressed relatively to untreated cells.

### 4.5. DNA Damage

DNA damage was determined by the alkaline comet assay as described previously [35]. The basic procedure was modified by the use of two repair enzymes—T4 pyrimidine dimer glycosylase, which recognized *cis-syn* cyclobutane pyrimidine dimers, and hOGG1, recognizing oxidatively modified DNA bases, including 8-oxoG. After UV irradiation and cell lysis, slides from the comet assay were washed with the respective enzymatic buffer and drained. The slides, covered by agarose, were soaked with enzyme buffer (40 mM HEPES, 0.1 M KCl, 0.5 mM EDTA, 0.2 mg/mL BSA, pH 8.0 with KOH) with or without 0.5 U T4 PDG or 0.16 U hOGG1 and incubated for 1 h at 37 °C and then processed according to comet assay procedure [36]. This allowed us to reveal UV-induced DNA damage, cyclobutane pyrimidine dimers and DNA bases oxidative modification, normally non-detected in the comet assay.

### 4.6. Reactive Oxygen Species

Intracellular ROS level was measured by 2′,7′-dichlorodihydrofluorescein as described previously [36].

### 4.7. Apoptosis

Annexin V-FITC Apoptosis Detection Kit I (BD Pharmingen, Heidelberg, Germany) was used according to the manufacturer’s instructions. Briefly, 1 × 10^5^ cells in 100 μL volume were mixed with 5 μL of FITC-conjugated annexin-V (annexin-V FITC) and 5 μL of propidium iodide (PI), vortexed gently, and incubated in the dark at room temperature for 15 min. A total of 400 μL of binding buffer was then added to each tube. The samples were then measured immediately with a Becton Dickinson cytometer, model LSRII (Becton Dickinson, San Jose, CA, USA). Emissions were measured in a separate fluorescent channel with doublet-discriminator module turned on. A laser excitation wavelength of 488 nm, using 530/30 nm band pass filter for the fluorescence parameter FL1 was used for FITC and a 585/42 nm band pass filter for FL2 for PI detection. Data were analyzed using FlowJo software (Tree Star Inc., Ashland, OR, USA).

The apoptosis was presented in the form of apoptotic index—a measure of the number of either early and late apoptotic cells plus necrotic cells or only early apoptotic cells expressed as a ratio of all cells counted.

### 4.8. Mitochondrial Membrane Potential

Mitochondrial membrane potential (MMP) was determined by MitoProbe™ JC-1 Assay Kit (Life Technologies, Grand Island, NY, USA) containing the cationic dye JC-1 (5′,6,6′-tetrachloro-1,1′,3,3′-tetraethylbenzimidazolylcarbocyanine iodide) and mitochondrial membrane potential disrupter CCCP (carbonyl cyanide 3 chlorophenylhydrazone) using fluorescence microplate reader and fluorescence microscopy. JC-1 accumulates in the mitochondrial membrane in a potential-dependent manner. High potential of the inner mitochondrial membrane facilitates formation of the dye aggregates (J-aggregates) with both excitation and emission shifted towards red light (530 nm/590 nm) when compared with that for JC-1 monomers (485 nm/538 nm). Cells were seeded into black 96-well tissue culture plates with transparent bottom (Greiner) at a density of 5 × 10^4^ cells/well in 100 µL culture medium and cultured in a CO_2_ incubator at 37 °C for 24 h. Each experiment included a positive control—10 μM of CCCP was added to the cells as an uncoupler of mitochondrial oxidation. The cells were preincubated with 5 μM JC-1 in the HBSS in a CO_2_ incubator at 37 °C for 30 min. Prior to measurement, the cells were centrifuged (300× *g* for 10 min at 22 °C) and washed twice with HBSS. Fluorescence was measured in Bio-Tek Synergy HT Microplate Reader (Bio-Tek Instruments, Winooski, VT, USA) with the filter pairs of 530 nm/590 nm and 485 nm/538 nm. Results are shown as a ratio of fluorescence, measured at 530 nm/590 nm (aggregates) to that measured at 485 nm/538 nm (monomers).

For fluorescence microscopy, cells were cultured on 6-well plate at a density of 1 × 10^6^ cells/well in 5000 µL culture medium, treated with or without UV and placed in a CO_2_ incubator at 37 °C for 24 h. At the end of the incubation, cells were stained with 10 µM JC-1 in HBSS and again incubated at 37 °C for 30 min. Next, the cells were centrifuged (300× *g* for 5 min at 23 °C) then washed twice and finally fixed in HBSS on microscope slides and visualized using IX70 Olympus inverted fluorescence microscope (Olympus, Tokyo, Japan) at 400× magnification.

### 4.9. Gene Expression

Total RNA was extracted from 6 × 10^6^ cells by using ISOLATE II RNA Mini Kit, according to the manufacturer’s instructions. RNA purity and concentration were determined by comparing the absorbances at 260 and 280 nm. The purified RNA samples were stored in TE buffer (5 mM Tris–HCl, 0.1 mM EDTA, pH 8.5 in DEPC-treated water), at −20 °C until further analysis.

cDNA was synthesized from total RNA using the High-Capacity cDNA Reverse Transcription Kit. A sample of 2 ng total RNA was used as a template in a total volume of 20 µL, following the manufacturer’s instructions. Gene expression were analyzed by TaqMan probe-based real-time PCR assay. TaqMan probes used for each gene are presented in Table 1. The reactions were carried out in a thermal cycler CFX96™ Real-Time PCR Detection System (BIO-RAD Laboratories, Hercules, CA, USA). The thermal cycling conditions were as follows: 10 min of polymerase activation at 95 °C, followed by 40 cycles of 30 s denaturation at 95 °C and 60 s annealing/extension at 60 °C. Each sample was run in duplicate. The cycle threshold (*C*_t_) values were calculated automatically by CFX96™ Real-Time PCR Detection System software (BIO-RAD). The basal expression level was calculated using the 2^−Δ*C*t^ model with *ACTB/RNR2* as an internal control [37].

**Table 1:** TaqMan probes used for RT-PCR gene expression assay.

Gene Name and Symbol	Assay ID
Beta actin (*ACTB*)	Mm00607939_s1
Mitochondrially encoded 16S ribosomal RNA (*RNR2*)	Mm04260181_s1
Mitochondrially encoded cytochrome b (*CYTB*)	Mm04225271_g1
Mitochondrially encoded cytochrome c oxidase subunit I (*COX1*)	Mm04225243_g1
Mitochondrially encoded NADH dehydrogenase subunit 3 (*ND3*)	Mm04225292_g1
Myeloid cell leukemia sequence 1 (*MCL-1*)	Mm01257351_g1
Succinate dehydrogenase complex, subunit B (*SDHB*)	Mm00458272_m1

### 4.10. Y253H and T315I Mutation Detection

RNA was isolated from 1 × 10^6^ cells by using the ISOLATE II RNA Mini Kit, according to the manufacturer’s recommendations. Next, cDNA was synthesized using 2 ng of total RNA and the High-Capacity cDNA Reverse Transcription Kit at the following temperatures: 10 min 25 °C, 120 min 37 °C and 5 min 85 °C. Conditions for allele-specific PCR amplifications were optimized on a thermal cycler CFX96™ Real-Time PCR Detection System (BIO-RAD) and the results were analyzed by using CFX Manager v 3.0 software (BIO-RAD Laboratories, Hercules, CA, USA).

RT-PCR reactions were performed using Luminaris HiGreen qPCR Master Mix (Thermo Fisher Scientific, West Palm Beach, FL, USA). Total reaction volume of 10 µL contained 1 μL of cDNA, 1× Master Mix, including SYBR Green I fluorescent dye, 0.3 µM each primer (Sigma-Aldrich, Hamburg, Germany). The PCR profile consisted of a UDG pre-treatment at 50 °C for 2 min, an initial denaturation step for 10 min at 95 °C, 50 cycles at 95 °C for 15 s, 60 s at 60 °C annealing/extension temperature. The following primers were used: sense–5′-ACTCCAGACTGTCCACAGCAT-3′ and allele specific antisense 5′-CGTAGGTCATGAACTCAA-3′ for T315I, and 5′-CGTACACCTCCCCGTG-3′ for Y253H.

### 4.11. Data Analysis

Statistical analyses were performed with SigmaPlot v11.0 software (Systat Software, Inc., San Jose, CA, USA). In general, *BCR-ABL1-*transfected imatinib-resistant cells were compared with their imatinib-sensitive counterparts. No comparison was made between BCR-ABL1-positive and parental, BCR-ABL1-negative cells. For cell viability assay and MMP, six replicates were analyzed for each cell line. For the RT-PCR results, the data represents the means of three replications. Statistical significance was determined by performing one-way ANOVA, with Tukey *post-hoc* multiple comparison or Student’s *t*-test.

## 5. Conclusions

In conclusion, imatinib-resistant cells may display different extents of genome instability than their imatinib-sensitive counterparts, which may results from/in their different reactions to both endogenous and exogenous DNA-damaging factors, especially DNA repair and apoptosis.

## References

[1] Chessum N., Jones K., Pasqua E., Tucker M. (2015). Recent advances in cancer therapeutics. Prog. Med. Chem..

[2] Rask-Andersen M., Zhang J., Fabbro D., Schiöth H.B. (2014). Advances in kinase targeting: Current clinical use and clinical trials. Trends Pharmacol. Sci..

[3] Jabbour E., Kantarjian H., Cortes J. (2015). Use of second- and third-generation tyrosine kinase inhibitors in the treatment of chronic myeloid leukemia: An evolving treatment paradigm. Clin. Lymphoma Myeloma Leuk..

[4] Balabanov S., Braig M., Brümmendorf T.H. (2014). Current aspects in resistance against tyrosine kinase inhibitors in chronic myelogenous leukemia. Drug Discov. Today Technol..

[5] Skorski T. (2008). BCR/ABL, DNA damage and DNA repair: Implications for new treatment concepts. Leuk. Lymphoma.

[6] Koptyra M., Cramer K., Slupianek A., Richardson C., Skorski T. (2008). BCR/ABL promotes accumulation of chromosomal aberrations induced by oxidative and genotoxic stress. Leukemia.

[7] Gaymes T.J., Mufti G.J., Rassool F.V. (2002). Myeloid leukemias have increased activity of the nonhomologous end-joining pathway and concomitant DNA misrepair that is dependent on the Ku70/86 heterodimer. Cancer Res..

[8] Koptyra M., Falinski R., Nowicki M.O., Stoklosa T., Majsterek I., Nieborowska-Skorska M., Blasiak J., Skorski T. (2006). BCR/ABL kinase induces self-mutagenesis via reactive oxygen species to encode imatinib resistance. Blood.

[9] Majsterek I., Sliwinski T., Poplawski T., Pytel D., Kowalski M., Slupianek A., Skorski T., Blasiak J. (2006). Imatinib mesylate (STI571) abrogates the resistance to doxorubicin in human K562 chronic myeloid leukemia cells by inhibition of BCR/ABL kinase-mediated DNA repair. Mutat. Res..

[10] Sliwinski T., Czechowska A., Szemraj J., Morawiec Z., Skorski T., Blasiak J. (2008). STI571 reduces NER activity in BCR/ABL-expressing cells. Mutat. Res..

[11] Stoklosa T., Poplawski T., Koptyra M., Nieborowska-Skorska M., Basak G., Slupianek A., Rayevskaya M., Seferynska I., Herrera L., Blasiak J. (2008). BCR/ABL inhibits mismatch repair to protect from apoptosis and induce point mutations. Cancer Res..

[12] Poplawski T., Blasiak J. (2010). BCR/ABL downregulates DNA-PK(CS)-dependent and upregulates backup non-homologous end joining in leukemic cells. Mol. Biol. Rep..

[13] Slupianek A., Falinski R., Znojek P., Stoklosa T., Flis S., Doneddu V., Pytel D., Synowiec E., Blasiak J., Bellacosa A. (2013). BCR-ABL1 kinase inhibits uracil DNA glycosylase UNG2 to enhance oxidative DNA damage and stimulate genomic instability. Leukemia.

[14] Nieborowska-Skorska M., Flis S., Skorski T. (2014). AKT-induced reactive oxygen species generate imatinib-resistant clones emerging from chronic myeloid leukemia progenitor cells. Leukemia.

[15] Reichrath J., Rass K. (2014). Ultraviolet damage, DNA repair and vitamin D in nonmelanoma skin cancer and in malignant melanoma: An update. Adv. Exp. Med. Biol..

[16] Kulms D., Zeise E., Pöppelmann B., Schwarz T. (2002). DNA damage, death receptor activation and reactive oxygen species contribute to ultraviolet radiation-induced apoptosis in an essential and independent way. Oncogene.

[17] Widel M., Krzywon A., Gajda K., Skonieczna M., Rzeszowska-Wolny J. (2014). Induction of bystander effects by UVA, UVB, and UVC radiation in human fibroblasts and the implication of reactive oxygen species. Free Radic. Biol. Med..

[18] Slupianek A., Hoser G., Majsterek I., Bronisz A., Malecki M., Blasiak J., Fishel R., Skorski T. (2002). Fusion tyrosine kinases induce drug resistance by stimulation of homology-dependent recombination repair, prolongation of G_2_/M phase, and protection from apoptosis. Mol. Cell. Biol..

[19] Tobin L.A., Robert C., Rapoprt A.P., Gojo I., Baer M.R., Tomkinson A.E., Rassol F.V. (2013). Targeting abnormal DNA double strand break repair in tyrosine kinase inhibitor-rfesistant chronic myeloid leukemias. Oncogene.

[20] Bolton-Gillespie E., Schemionek M., Klein H.U., Flis S., Hoser G., Lange T., Nieborowska-Skorska M., Maier J., Kerstiens L., Koptyra M. (2013). Genomic instability may originate from imatinib-refractory chronic myeloid leukemia stem cells. Blood.

[21] Mouret S., Philippe C., Gracia-Chantegrel J., Banyasz A., Karpati S., Markovitsi D., Douki T. (2010). UVA-induced cyclobutane pyrimidine dimers in DNA: A direct photochemical mechanism?. Org. Biomol. Chem..

[22] Canitrot Y., Falinski R., Louat T., Laurent G., Cazaux C., Hoffmann J.S., Lautier D., Skorski T. (2003). p210 BCR/ABL kinase regulates nucleotide excision repair (NER) and resistance to UV radiation. Blood.

[23] Guillem V.M., Cervantes F., Martínez J., Alvarez-Larrán A., Collado M., Camós M., Sureda A., Maffioli M., Marugán I., Hernández-Boluda J.C. (2010). XPC genetic polymorphisms correlate with the response to imatinib treatment in patients with chronic phase chronic myeloid leukemia. Am. J. Hematol..

[24] Sugasawa K. (2010). Regulation of damage recognition in mammalian global genomic nucleotide excision repair. Mutat. Res..

[25] Dinis J., Silva V., Gromicho M., Martins C., Laires A., Tavares P., Rendeiro P., Torres F., Rueff J., Rodriguez A. (2012). DNA damage response in imatinib resistant chronic myeloid leukemia K562 cells. Leuk. Lymphoma.

[26] Zhivotovsky B., Kroemer G. (2004). Apoptosis and genomic instability. Nat. Rev. Mol. Cell Biol..

[27] Surova O., Zhivotovsky B. (2013). Various modes of cell death induced by DNA damage. Oncogene.

[28] Desplat V., Belloc F., Lagarde V., Boyer C., Melo J.V., Reiffers J., Praloran V., Mahon F.X. (2005). Overproduction of BCR-ABL induces apoptosis in imatinib mesylate-resistant cell lines. Cancer.

[29] Gottlieb E., Armour S.M., Harris M.H., Thompson C.B. (2003). Mitochondrial membrane potential regulates matrix configuration and cytochrome *c* release during apoptosis. Cell. Death Differ..

[30] Grimm S. (2013). Respiratory chain complex II as general sensor for apoptosis. Biochim. Biophys. Acta.

[31] Huang C.-R., Yang-Yen H.-F. (2010). The fast-mobility isoform of mouse Mcl-1 is a mitochondrial matrix-localized protein with attenuated anti-apoptotic activity. FEBS Lett..

[32] Wang Z., Liu Z., Wu X., Chu S., Wang J., Yuan H., Roth M., Yuan Y.-C., Bhatia R., Chen W.Y. (2014). ATRA-induced cellular differentiation and CD38 expression inhibits acquisition of BCR-ABL mutations for CML acquired resistance. PLoS Genet..

[33] Bixby D., Talpaz M. (2011). Seeking the causes and solutions to imatinib-resistance in chronic myeloid leukemia. Leukemia.

[34] Jabbour E., Kantarjian H. (2014). Chronic myeloid leukemia: 2014 update on diagnosis, monitoring, and management. Am. J. Hematol..

[35] Pawlowska E., Wysokiński D., Tokarz P., Piastowska-Ciesielska A., Szczepanska J., Blasiak J. (2014). Dexamethasone and 1,2,5-dihydroxyvitamin D3 reduce oxidative stress-related DNA damage in differentiating osteoblasts. Int. J. Mol. Sci..

[36] Collins A., Harrington V. (2002). Repair of oxidative DNA damage: Assessing its contribution to cancer prevention. Mutagenesis.

[37] Schmittgen T.D., Livak K.J. (2008). Analyzing real-time PCR data by the comparative *C*_t_ method. Nat. Protoc..

